# Elastoplastic Model Framework for Saturated Soils Subjected to a Freeze–Thaw Cycle Based on Generalized Plasticity Theory

**DOI:** 10.3390/ma14216485

**Published:** 2021-10-28

**Authors:** Shengyi Cong, Xianzhang Ling, Xinyu Li, Lin Geng, Wenqiang Xing, Guoyu Li

**Affiliations:** 1 School of Civil Engineering, Harbin Institute of Technology, Harbin 150090, China; lingxianzhang@hit.edu.cn (X.L.); 18B933025@stu.hit.edu.cn (X.L.); xingwenqiang@hit.edu.cn (W.X.); 2 State Key Laboratory of Frozen Soil Engineering, Northwest Institute of Eco-Environment and Resources, China Academy of Sciences, Lanzhou 730000, China; guoyuli@lzb.ac.cn; 3 Chongqing Research Institute, Harbin Institute of Technology, Chongqing 401135, China; 4 Railway Engineering Research Institute, China Academy of Railway Sciences Corporation Limited, Beijing 100081, China; 12B333004@hit.edu.cn

**Keywords:** constitutive relation, generalized plasticity theory, double yield surfaces, saturated soils, freeze–thaw cycle

## Abstract

The failures of soil slopes during the construction of high-speed railway caused by the soil after the freeze–thaw (F–T) cycle and the subsequent threat to construction safety are critical issues. An appropriate constitutive model for soils accurately describing the deformation characteristics of soil slopes after the F–T cycle is very important. Few constitutive models of soils incorporate the F–T cycle, and the associated flow rule has always been employed in previous models, which results in an overestimation of the deformation of soil exposed to the F–T cycle. Generalized plasticity theory is widely used to predict the performance of geotechnical materials and is especially well adapted to deal with this type of generalized cyclic loading (such as a freeze–thaw cycle), and it overcomes the shortcomings of the associated flow rule that causes larger shear deformation. To this end, an elastoplastic model framework based on generalized plasticity theory with double yield surfaces for saturated soils subjected to F–T cycles was developed. Two types of plastic deformation mechanisms, i.e., plastic volumetric compression and plastic shear, were considered in this elastoplastic model. It was found that this model can accurately predict the mechanical behavior and deformation characteristics of saturated soils after F–T cycles.

## 1. Introduction

**Figure 1:**
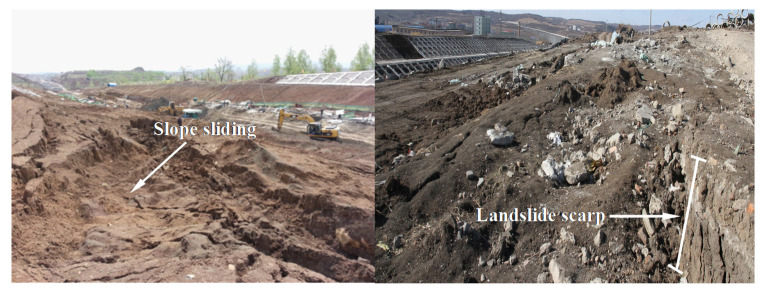
The cutting slope failures along the high-speed railway (the left figure is from [14]).

High-speed railways in China have developed rapidly over the last decade [1]. By the end of 2021, the total length of the high-speed railway systems in China is expected to be more than 40,000 km. A high-speed railway has been constructed in a typical seasonally frozen region of Northeast China [2,3]. The freeze–thaw (F–T) cycle is one of the most common physical weathering processes in these regions [4] and significantly affects the mechanical behavior of soils [5,6,7,8,9,10,11,12,13]. Some studies have reported that F–T cycles induce cutting slope failures (Figure 1) [14] during the construction of high-speed railway. An appropriate constitutive model for soils that accurately describes the deformation characteristics of soil slope after the F–T cycle is very important.

Recently, many studies have been carried out to analyze the effect of wet/dry cycles on the deformation behavior and constitutive stress–strain of soil [15,16,17,18,19]. Furthermore, a number of researchers, such as Li et al. 2019 [20], Li and Yang, 2018 [21], Guo et al. 2021 [22], and Li et al. 2020 [23], have studied the elastoplastic model of unsaturated soils, incorporating different influencing factors. However, the literature about the constitutive model of saturated soils considering the effect of the F–T cycle is relatively sparse. Furthermore, the associated flow rule was usually adopted in the constitutive model of soils [24,25]. The traditional associated flow rule always induces larger shear deformation [26,27], which overestimates the deformation of soil slopes exposed to the F–T cycle.

Generalized plasticity theory is widely used to predict the performances of geotechnical materials [28,29]. The effect of freezing–thawing cycles on the behavior of soils is a matter of great practical importance. Generalized plasticity is especially well adapted to deal with this type of generalized cyclic loading [29]. The generalized plasticity theory does not obey the traditional assumption of plastic potential function, nor does it comply with the associated plastic flow rule [28]. It overcomes the shortcomings of the associated flow rule causing larger shear deformation. As a result, the use of the framework of generalized plasticity theory is particularly useful to evaluate the mechanical behavior of soil. However, few models for soils subjected to the F–T cycle have been based on the generalized plasticity theory.

In this study, an elastoplastic model for saturated soils subjected to F–T cycles under the framework of generalized plasticity theory with double yield surfaces was developed. The derivation of the model and the estimation of the model parameters are presented in detail. The triaxial test results of the saturated soils were predicted by the proposed model, and finally, the effectiveness of the model was verified using the test data.

## 2. An Elastoplastic Model Framework for Saturated Soils in Generalized Plasticity Theory Incorporating the Effects of the Freeze–Thaw Cycle

An elastoplastic model framework for saturated soils subjected to F–T cycles was developed using the generalized plasticity theory. Moreover, a detailed introduction to the generalized plasticity theory is presented in the Appendix A.

### 2.1. Elastic Deformations

In this section, the elastic deformations are discussed, and the soil is regarded as isotropic and elastic within the yield surface. The increments in the strain invariants consist of two parts [30], i.e.,

(1) $$d\varepsilon_{s}=d\varepsilon_{s}^{e}+d\varepsilon_{s}^{p}$$

(2) $$d\varepsilon_{v}=d\varepsilon_{v}^{e}+d\varepsilon_{v}^{p}$$

where d*ε*_s_, d$\varepsilon_{s}^{e}$, and d$\varepsilon_{s}^{p}$ are the increment of shear strain, elastic shear strain, and plastic shear strain, respectively; and d*ε*_v_, d$\varepsilon_{v}^{e}$, and d$\varepsilon_{v}^{p}$ are the increment of volumetric strain, elastic volumetric strain, and plastic volumetric strain, respectively.

The elastic components of the strain increments (d$\varepsilon_{s}^{e}$ and d$\varepsilon_{v}^{e}$) can be obtained from

(3) $$d\varepsilon_{s}^{e}=\frac{1}{3G}\;dq$$

(4) $$d\varepsilon_{v}^{e}=\frac{1}{K}\;dp^{\prime }$$

where *p*′ and *q* are the mean effective stress and deviator stress, respectively; *p*′ *=* $\frac{1}{3}$(*σ*′_1_ *+σ*′_2_ + *σ*′_3_), *q = σ*′_1_ − *σ*′_3_. *σ*′_1_, *σ*′_2_, and *σ*′_3_ are the major principal effective stress, the intermediate principal effective stress, and the minor principal effective stress, respectively. *G* and *K* are the shear modulus and bulk modulus, respectively, and can be deduced from the elastic modulus (*E*) for an assumed value of Poisson’s ratio (*v*):

(5) $$G=\frac{E}{2(1+v)}$$

(6) $$K=\frac{E}{3(1\;-\;2v)}\;$$

### 2.2. Yield Surfaces and Plastic Potential Functions

The deformation of soil slope after the F–T cycle, which includes the shear deformation, compression deformation, or a combination of the two deformations, is complicated. The double yield surfaces proposed by Yin (1988) [31] could reflect two types of plastic deformation mechanisms, namely, plastic volumetric compression and plastic shear for soils, and it is often employed by researchers [24,32] to present the mechanical and deformation characteristics of soils. Therefore, the double yield surfaces proposed by Yin (1988) [19] were used in this paper.

**Figure 2:**
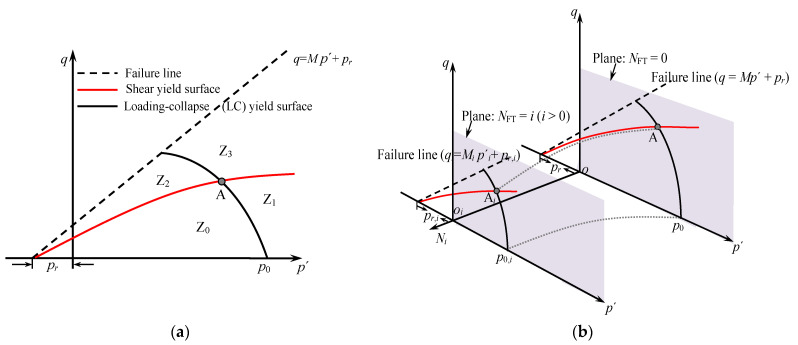
Yield surfaces in *q*-*p*′ space: (**a**) Yin’s proposed yield surfaces (1988); (**b**) yield surfaces under freeze–thaw cycles.

Figure 2a shows the two yield surfaces proposed by Yin (1988) [31] in the *q*-*p*′ plane. Point A is the intersection of the two yield surfaces. Referring to the yield surfaces in [31], the yield surfaces of soils subjected to F–T cycling are plotted in Figure 2b. Two yield surfaces divide the *q*-*p*′ plane into four parts [31]: region Z_0_ only has elastic deformation, region Z_1_ is only related to the first yield surface, region Z_2_ is only related to the second yield surface, and the two kinds of plastic deformation exist simultaneously in region Z_3_.

In the first yield surface (*f*_v_), the loading–collapse (LC) yield surface represents the compression mechanism and is expressed as the following form:

(7) $$f_{v}(p^{\prime },\;q,\;\varepsilon_{v}^{p})=p^{\prime }+\frac{q^{2}}{{(p^{\prime }+p}_{r}{)M}_{1}^{2}}-p_{0}$$

where *M*_1_, *p*_0_, and *p*_r_ are the LC yield surface indexes that are related to the shape of the stress–strain curve of soils, the initial mean effective stress, and the intercept of the failure line on the *p*′ axis, respectively.

In addition, symbols *M_i_*, *p*′*_i_*, *p*_r__,*i*_, and *p*_0,*i*_ are the parameters under various numbers of freeze–thaw cycles (*N*_FT_), which are the slope of failure line, mean effective stress, intercept of the failure line on the *p*′ axis, and initial mean effective stress, respectively. Subscript *i* represents the number of freeze–thaw cycles (*i* = 0, 1, 3, 7, and 11), which is the same meaning as *N*_FT_. For convenience, the symbol *i* was used in this section.

A hyperbolic curve is used to predict the relation between plastic volumetric strain ($\varepsilon_{v}^{p}$) and *p*_0_ [31]:

(8) $$p_{0}=\frac{\chi_{1}\varepsilon_{v}^{p}}{{1-\chi }_{2}\varepsilon_{v}^{p}}\;p_{a}$$

where *χ*_1_, *χ*_2_, and *p*_a_ are the elastoplastic compression indexes and air pressure, respectively.

The $\varepsilon_{v}^{p}\;$ was chosen as the hardening parameter. A non-associated plastic flow rule was adopted in this model. Referring to [33], the mean effective stress *p*′ is assumed to be the plastic potential function (*Q*_v_) corresponding to the plastic volumetric strain.

*Q*_v_ = *p*′ (9)

The second yield surface (*f*_q_) proposed by Yin (1988) [31] is employed to present the shearing mechanism of the soils:

(10) $$f_{q}(p^{\prime },\;q,\;\varepsilon_{s}^{p})=\frac{aq}{G}\sqrt{\frac{q}{M_{2}\left({p^{\prime }+p}_{r}\right)-q}}\;-\varepsilon_{s}^{p}$$

where *a* and *M*_2_ are the elastoplastic dilation index and shear yield surface index that is related to the slope of failure line (*M*), respectively. $\varepsilon_{s}^{p}$ is the plastic shear strain.

Similarly, $\varepsilon_{s}^{p}$ is used as the hardening parameter for the second yield surface. Referring to [33], the plastic potential function (*Q*_q_) is assumed to be *q*, corresponding to the plastic shear strain.

*Q*_q_ = *q* (11)

### 2.3. Elastoplastic Stress–Strain Relations

The incremental stress–strain relationship of soils in *p*′ − *q* plane can be obtained [28]:

(12) $$\left\{d\sigma \right\}=[D_{e}]\;\left\{d\varepsilon \right\}-[D_{e}]d\lambda_{v}\left\{\frac{\partial Q_{v}}{\partial \sigma }\right\}-[D_{e}]d\lambda_{q}\left\{\frac{\partial Q_{q}}{\partial \sigma }\right\}$$

where d*λ*_v_ and d*λ*_q_ are the plastic factors (see Appendix A). The elastic stiffness matrix, [*D*_e_], can be determined by Equation (13):

(13) $$[D_{e}]=\left[\begin{array}{cc} K & 0\\ 0 & 3G \end{array}\right]$$

Based on Equations (9), (11), (12), (A12), and (A13), the following expressions can be obtained [28]:

(14) $$\left(1+\frac{1}{A_{v}}{\left\{\frac{\partial f_{v}}{\partial \sigma }\right\}}^{T}\left[D_{e}\right]\left\{\frac{\partial p’}{\partial \sigma }\right\}\right)d\lambda_{v}=\frac{1}{A_{v}}{\left\{\frac{\partial f_{v}}{\partial \sigma }\right\}}^{T}\left[D_{e}\right]\left\{d\varepsilon \right\}-\frac{1}{A_{v}}{\left\{\frac{\partial f_{v}}{\partial \sigma }\right\}}^{T}\left[D_{e}\right]d\lambda_{q}\left\{\frac{\partial q}{\partial \sigma }\right\}$$

(15) $$\left(1+\frac{1}{A_{q}}{\left\{\frac{\partial f_{q}}{\partial \sigma }\right\}}^{T}\left[D_{e}\right]\left\{\frac{\partial q}{\partial \sigma }\right\}\right)d\lambda_{q}=\frac{1}{A_{q}}{\left\{\frac{\partial f_{q}}{\partial \sigma }\right\}}^{T}\left[D_{e}\right]\left\{d\varepsilon \right\}-\frac{1}{A_{q}}{\left\{\frac{\partial f_{q}}{\partial \sigma }\right\}}^{T}\left[D_{e}\right]d\lambda_{v}\left\{\frac{\partial p’}{\partial \sigma }\right\}$$

where *A*_v_ and *A*_q_ are the plastic parameters.

The related parameters are given by

(16) $$\frac{\partial f_{v}}{\partial p’}=1-\frac{q^{2}}{M_{1}^{2}{\left(p{’+p}_{r}\right)}^{2}}$$

(17) $$\frac{\partial f_{v}}{\partial q}=\frac{2q}{M_{1}^{2}\left(p{’+p}_{r}\right)}$$

(18) $$\frac{\partial f_{v}}{\partial \varepsilon_{v}^{p}}=\frac{\chi_{1}p_{a}}{{\left(1-\chi_{2}\varepsilon_{v}^{p}\right)}^{2}}$$

(19) $$\frac{\partial f_{q}}{\partial p’}=-\frac{{aq}^{2}M_{2}}{2G}\times \frac{1}{\sqrt{\frac{q}{M_{2}\left(p{’+p}_{r}\right)-q}}}\times \left\{\frac{1}{{\left[M_{2}\left(p{’+p}_{r}\right)-q\right]}^{2}}\right\}$$

(20) $$\frac{\partial f_{q}}{\partial p’}=\frac{a}{G}\sqrt{\frac{q}{M_{2}\left(p{’+p}_{r}\right)-q}}+\frac{aq}{2G}\times \frac{1}{\sqrt{\frac{q}{M_{2}\left(p{’+p}_{r}\right)-q}}}\times \frac{M_{2}\left(p{’+p}_{r}\right)}{{\left[M_{2}\left(p{’+p}_{r}\right)-q\right]}^{2}}$$

(21) $$\frac{\partial f_{q}}{\partial \varepsilon_{s}^{p}}=1$$

(22) $$\left\{d\sigma \right\}=\left\{\begin{array}{c} dp’\\ dq \end{array}\right\}$$

(23) $$\left\{d\varepsilon \right\}=\left\{\begin{array}{c} {d\varepsilon }_{v}\\ {d\varepsilon }_{q} \end{array}\right\}$$

The elastoplastic incremental stress–strain relationship can be given by

(24) $$\left\{d\sigma \right\}=[D_{\mathrm{ep}}]\;\left\{d\varepsilon \right\}$$

According to [28], the elastoplastic stiffness matrix [*D*_ep_] takes the following form:

(25) $$[D_{\mathrm{ep}}]=[D_{e}]-\frac{\left[D_{e}\right]\left(w_{1}{+w}_{2}\right)\left[D_{e}\right]}{A_{v}A_{q}{+A}_{v}t_{4}{+A}_{q}t_{1}{+t}_{1}t_{4}{-t}_{3}t_{6}}$$

where *w*_1_ and *w*_2_ are given by

(26) $$w_{1}=\left\{\frac{\partial p’}{\partial \sigma }\right\}\left[\left(A_{q}{+t}_{4}\right){\left\{\frac{\partial f_{v}}{\partial \sigma }\right\}}^{T}-t_{3}{\left\{\frac{\partial f_{q}}{\partial \sigma }\right\}}^{T}\right]$$

(27) $$w_{2}=\left\{\frac{\partial q}{\partial \sigma }\right\}\left[\left(A_{v}{+t}_{1}\right){\left\{\frac{\partial f_{q}}{\partial \sigma }\right\}}^{T}-t_{6}{\left\{\frac{\partial f_{v}}{\partial \sigma }\right\}}^{T}\right]\;$$

where *t*_1_, *t*_2_, *t*_3_, *t*_4_, *t*_5_, and *t*_6_ can be obtained by Equations (A16)–(A21) (see Appendix A).

## 3. Determination of Parameters and Model Validation

The parameters employed in the model fall into three categories: elastic parameters, parameters related to the compressive mechanism, and parameters related to the shear mechanism. The model was employed to predict the test data for soils after the F–T cycle.

### 3.1. Elastic Parameters

The elastic parameters (*G* and *K*) can be determined from triaxial compressive loading–unloading–reloading testing. Moreover, *G* and *K* can also be obtained from Equations (5) and (6), whereas the value of Poisson’s ratio (*v*) equal to 0.3 is assumed in this model [34,35].

### 3.2. Parameters Related to a Compressive Mechanism

The LC yield surface index, *M*_1_, which is related to the shape of stress–strain curve, is calculated through the following equation [31]:

*M*_1_ = (1 + 0.25*β*^2^)*M* (28)

where *β* is the ratio of the volumetric strain (*ε*_v_) to axial strain (*ε*_a_) when the stress level equals 75%; the mean value was used in the model under different confining pressure (*σ*_c_) and various *N*_FT_ values. *M* is the slope of the failure line, and it is also related to *N*_FT_.

The value of *p*_r_ can be obtained from the failure line under various *N*_FT_ values. The elastoplastic compression indexes (*χ*_1_ and *χ*_2_) have been reported by Yin (1988) [31] and will not be repeated here. However, a material parameter *Γ* was added in the equation $\sqrt{\Gamma \frac{B_{p}}{p_{a}}}$ (see Yin 1988 [31]) to represent the material characteristics (it is related to volumetric compression deformation, Γ = 2.5–3.0).

### 3.3. Parameters Related to a Shear Mechanism

The shear yield surface index, *M*_2_, which is related to the shape of the *q*-deviatoric strain (*ε*_s_) curve, can be estimated by the following equation [31]:

(29) $$M_{2}=M\;/\;R_{f}^{0.25}$$

where *R*_f_ is the failure ratio, which is defined by

*R*_f_ = *q*_f_ / *q*_ult_ (30)

where *q*_f_ and *q*_ult_ are the failure strength and the asymptotic value of deviatoric stress for the *q* − *ε*_s_ curve, respectively.

The elastoplastic dilation index (*a*) reflects the proportion of the strain induced by the shear yield surface in terms of the total strain. It can be estimated by the equation proposed by Yin (1988) (*a* = 0.25 − 0.15*d*, *d* is the slope of the *ε*_v_ − *ε*_a_ curve at a stress level between 75% and 95%).

In summary, two elastic parameters (*G* and *K*), four parameters (*M*_1_, *p_r_*, *χ*_1_, and *χ*_2_) related to the compressive mechanism, and two parameters (*M*_2_ and *a*) related to the shear mechanism were used in the proposed model. These eight parameters change along *N*_FT_ and they are all functions of *N*_FT_. These parameters can be obtained by test results under different *N*_FT_ values and empirical formulas.

### 3.4. Model Validation

**Figure 3:**
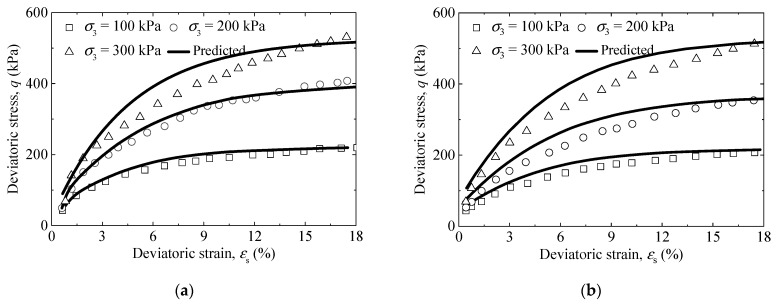
Comparison between predicted and observed results for stress–strain curves of saturated silty clay (data from [36]): (**a**) *N*_FT_ = 3 and (**b**) *N*_FT_ = 5.

**Figure 4:**
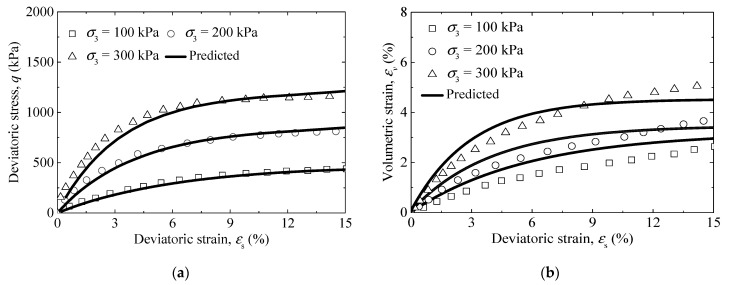
Comparison between predicted and observed results: (**a**) stress–strain curve, and (**b**) volumetric–deviatoric strain curve of bioenzyme-treated saturated soils (data from [37]).

The influence of the F–T cycle on the mechanical properties of silty clay was investigated by Cui et al. (2015) [36]. The triaxial test results of saturated silty clay exposed to three and five F–T cycles were used to verify the correctness of the model. The volumetric strain was measured under consolidation and shearing in [36]. The volumetric strain induced by consolidation was not given, so the volumetric strain during shearing could not be obtained. Therefore, only the stress–strain curves of the saturated silty clay were employed to verify the model’s correctness. The model parameters used in [36] for the third and fifth F–T cycle are shown in Table 1 and Table 2. Figure 3 plots the comparison between the experimental data and predicted results. From Figure 3, considering some parameter determination based on the empirical formula, the predicted values are within reasonable range, although there are some differences. In addition, the triaxial test results of bioenzyme-treated saturated soils with 3% bioenzyme from Wen and Wang (2018) [37] were also used to verify the model’s correctness. (although the bioenzyme-treated saturated soils are not subjected to the F–T cycle, the validation results can be used as proof that the model framework can be widely used). Model parameters used in [37] are given in Table 3. Form Figure 4, the stress–strain curve and volumetric–deviatoric strain curve of bioenzyme-treated saturated soils were reasonably predicted by the proposed model.

## 4. Future Fields of Application

This study presents an elastoplastic model framework for saturated soils subjected to the freeze–thaw cycle and also serves as a simple methodology to establish the constitutive model for soils. Two types of plastic deformation mechanisms, i.e., plastic volumetric compression and plastic shear, were considered in this elastoplastic model. Therefore, this model could be employed to analyze the relatively complex deformations of engineering, such as soil slope, railway roadbed, dam, and building foundation. In addition, the analysis discussed in this paper is mainly used to describe the deformation characteristics of strain hardening for clay soils. In practical applications, this important aspect must be given proper attention. Further study is needed for a more quantitative description of the strain softening of soils.

## 5. Conclusions

In this study, an elastoplastic model framework for saturated soils subjected to the freeze–thaw cycle based on the generalized plasticity theory was proposed. The primary conclusions from this work can be given as follows:

(1) This model was on the framework of the generalized plasticity theory overcoming the disadvantage of the traditionally associated flow rule, inducing larger shear deformation. The model adequately describes the contractive shear behavior of saturated soils under different freeze–thaw cycles.

(2) The capabilities of this model are illustrated by predicting the triaxial tests of silty clay and bioenzyme-treated soils. In this model, the overall elastoplastic model behaviors of saturated soils are described through eight model parameters that are specifically associated with the freeze–thaw cycle.

Further work will focus on the derivation of the elastoplastic constitutive model of soil after the freeze–thaw cycle in a real triaxial stress state and consider unsaturated conditions and more general stress paths.

## Figures and Tables

**Table 1:** Parameter values for the proposed elastoplastic model with *N*_FT_ = 3 used in [36].

Parameters	Values		
	*σ*_c_ = 100 kPa	*σ*_c_ = 200 kPa	*σ*_c_ = 300 kPa
Shear modulus, *G* (kPa)	2223	3359	4668
Bulk modulus, *K* (kPa)	4816	7280	10,114
Slope of failure line, *M*	1.0		
Shear yield surface index, *M*_2_	1.04		
Intercept of the failure line on the *p* axis, *p*_r_	42		
Elastoplastic dilation index, *a*	0.23		
Poisson’s ratio, *v*	0.3		

**Table 2:** Parameter values for the proposed elastoplastic model with *N*_FT_ = 5 used in [36].

Parameters	Values		
	*σ*_c_ = 100 kPa	*σ*_c_ = 200 kPa	*σ*_c_ = 300 kPa
Shear modulus, *G* (kPa)	1990	3108	4617
Bulk modulus, *K* (kPa)	4312	6734	10,003.5
Slope of failure line, *M*	0.98		
Shear yield surface index, *M*_2_	1.02		
Intercept of the failure line on the *p* axis, *p*_r_	38		
Elastoplastic dilation index, *a*	0.23		
Poisson’s ratio, *v*	0.3		

**Table 3:** Parameter values for the proposed elastoplastic model used in [37].

Parameters	Values		
	*σ*_c_ = 100 kPa	*σ*_c_ = 200 kPa	*σ*_c_ = 300 kPa
Shear modulus, *G* (kPa)	3901	11,194	18,867
Bulk modulus, *K* (kPa)	8452	24,253	40,879
Slope of failure line, *M*	1.6		
Loading–collapse (LC) yield surface index, *M*_1_	1.7		
Shear yield surface index, *M*_2_	1.71		
Intercept of the failure line on the *p* axis, *p*_r_	22.9		
Elastoplastic compression index, *χ*_1_	303.8		
Elastoplastic compression index, *χ*_2_	25.8		
Elastoplastic dilation index, *a*	0.23		
Poisson’s ratio, *v*	0.3		

## Data Availability

Not applicable.

## Appendix A. Theoretical Framework of Generalized Plasticity Theory

Generalized plasticity theory is widely used to analyze the behavior of geotechnical materials [28]. According to the theory of plasticity, the strain increment (d*ε_ij_*) is usually divided into an elastic component (d$\varepsilon_{\mathrm{ij}}^{e}$) and a plastic component (d$\varepsilon_{\mathrm{ij}}^{p}$) [28]:

(A1) $$d\varepsilon_{\mathrm{ij}}=d\varepsilon_{\mathrm{ij}}^{e}+d\varepsilon_{\mathrm{ij}}^{p}$$

The generalized potential theory can be expressed in the following form [28,33]:

(A2) $${d\varepsilon }_{\mathrm{ij}}^{p}=\overset{3}{\underset{k=1}{\sum }}{d\lambda }_{k}\frac{\partial Q_{k}}{\partial \sigma_{\mathrm{ij}}}\;\;$$

where *Q_k_* is the plastic potential function. *Q*_1_, *Q*_2_, and *Q*_3_ should be non-associated. d*λ_k_* and *σ_ij_* are the plastic factors corresponding to the plastic potential function and stress increments, respectively.

The yield surfaces should be coincident with the plastic potential surface [28]. The volumetric yield function *f*_v_ (*p*′, *q*, *θ*_σ_, *h*_v_), the shear yield function *f*_q_ (*p*′, *q*, *θ*_σ_, *h*_q_) in the *q*-direction, and the shear yield function *f*_θ_ (*p*′, *q*, *θ*_σ_, *h*_θ_) in the *θ*_σ_-direction take the following form [28]:

(A3) $$f_{v}(\sigma_{\mathrm{ij}},\;\varepsilon_{\mathrm{ij}}^{p})=fv_{\;}(p^{\prime },\;q,\;\theta_{\sigma },\;h_{v})=0$$

(A4) $$f_{q}(\sigma_{\mathrm{ij}},\;\varepsilon_{\mathrm{ij}}^{p})=f(p^{\prime },\;q,\;\theta_{\sigma },\;h_{q})=0$$

(A5) $$f_{\theta }(\sigma_{\mathrm{ij}},\;\varepsilon_{\mathrm{ij}}^{p})=f_{\theta }(p^{\prime },\;q,\;\theta_{\sigma },\;h_{\theta })=0$$

Differentiating Equations (A3)–(A5) and combining Equation (A2) gives [28]

(A6) $$d\lambda_{v}=\frac{1}{A_{v}}\frac{\partial f_{v}}{\partial p’}dp\prime +\frac{1}{A_{v}}\frac{\partial f_{v}}{\partial q}dq+\frac{1}{A_{v}}\frac{\partial f_{v}}{\partial \theta_{\sigma }}d\theta_{\sigma }$$

(A7) $$d\lambda_{q}=\frac{1}{A_{q}}\frac{\partial f_{q}}{\partial p’}dp\prime +\frac{1}{A_{q}}\frac{\partial f_{q}}{\partial q}dq+\frac{1}{A_{q}}\frac{\partial f_{q}}{\partial \theta_{\sigma }}d\theta_{\sigma }$$

(A8) $$d\lambda_{\theta }=\frac{1}{A_{\theta }}\frac{\partial f_{\theta }}{\partial p’}dp\prime +\frac{1}{A_{\theta }}\frac{\partial f_{\theta }}{\partial q}dq+\frac{1}{A_{\theta }}\frac{\partial f_{\theta }}{\partial \theta_{\sigma }}d\theta_{\sigma }$$

The plastic parameters, *A*_v_, *A*_q_, and *A*_θ_ can be given by [28,33]

(A9) $$A_{v}=-\frac{\partial f_{v}}{\partial h_{v}}{\left\{\frac{\partial h_{v}}{\partial \varepsilon^{p}}\right\}}^{T}\left\{\frac{\partial Q_{v}}{\partial \sigma }\right\}$$

(A10) $$A_{q}=-\frac{\partial f_{q}}{\partial h_{q}}{\left\{\frac{\partial h_{q}}{\partial \varepsilon^{p}}\right\}}^{T}\left\{\frac{\partial Q_{q}}{\partial \sigma }\right\}\;$$

(A11) $$A_{\theta }=-\frac{\partial f_{\theta }}{\partial h_{\theta }}{\left\{\frac{\partial h_{\theta }}{\partial \varepsilon^{p}}\right\}}^{T}\left\{\frac{\partial Q_{\theta }}{\partial \sigma }\right\}\;$$

where *h*_v_, *h*_q_, and *h*_θ_ are the hardening parameters.

The effect of *θ*_σ_ is very small and can be ignored [28,33]. Hence, Equations (A6)–(A8) can be rewritten as

(A12) $$d\lambda v_{\;}=\frac{1}{A_{v}}\frac{\partial f_{v}}{\partial p’}dp\prime +\frac{1}{A_{v}}\frac{\partial f_{v}}{\partial q}dq$$

(A13) $$d\lambda q_{\;}=\frac{1}{A_{q}}\frac{\partial f_{q}}{\partial p’}dp\prime +\frac{1}{A_{q}}\frac{\partial f_{q}}{\partial q}dq$$

The plastic factors d*λ*_v_ and d*λ*_q_ are computed as follows [28]:

(A14) $$d\lambda_{v}\;=\;\frac{A_{q}t_{2}{+t}_{2}t_{4}{-t}_{3}t_{5}}{A_{v}A_{q}{+A}_{v}t_{4}{+A}_{q}t_{1}{+t}_{1}t_{4}{-t}_{3}t_{6}}$$

(A15) $$d\lambda_{q}\;=\;\frac{A_{v}t_{5}{+t}_{1}t_{5}{-t}_{2}t_{6}}{A_{v}A_{q}{+A}_{v}t_{4}{+A}_{q}t_{1}{+t}_{1}t_{4}{-t}_{3}t_{6}}$$

where *t*_1_, *t*_2_, *t*_3_, *t*_4_, *t*_5_, and *t*_6_ can be expressed as [28]

(A16) $$t_{1}\;=\;{\left\{\frac{\partial f_{v}}{\partial \sigma }\right\}}^{T}\left[D_{e}\right]\left\{\frac{\partial p’}{\partial \sigma }\right\}$$

(A17) $$t_{2}\;=\;{\left\{\frac{\partial f_{v}}{\partial \sigma }\right\}}^{T}\left[D_{e}\right]\left\{d\varepsilon \right\}\;$$

(A18) $$t_{3}\;=\;{\left\{\frac{\partial f_{v}}{\partial \sigma }\right\}}^{T}\left[D_{e}\right]\left\{\frac{\partial q}{\partial \sigma }\right\}\;$$

(A19) $$t_{4}\;=\;{\left\{\frac{\partial f_{q}}{\partial \sigma }\right\}}^{T}\left[D_{e}\right]\left\{\frac{\partial q}{\partial \sigma }\right\}\;$$

(A20) $$t_{5}\;=\;{\left\{\frac{\partial f_{q}}{\partial \sigma }\right\}}^{T}\left[D_{e}\right]\left\{d\varepsilon \right\}\;$$

(A21) $$t_{6}\;=\;{\left\{\frac{\partial f_{q}}{\partial \sigma }\right\}}^{T}\left[D_{e}\right]\left\{\frac{\partial p’}{\partial \sigma }\right\}\;$$
